# The use of bedside case-based learning in the clinical practice of midwifery education in China

**DOI:** 10.1186/s12909-024-06251-y

**Published:** 2024-11-14

**Authors:** Yao Zhang, Xinfen Xu, Fang Wang, Lewei Tu, Qinqi Deng, Mengyan Xu, Guijuan He, Linda Johnston

**Affiliations:** 1 School of Nursing, Hangzhou Normal University, Hangzhou City, Zhejiang Province China; 2 School of Nursing, Zhejiang Chinese Medicine University, Hangzhou City, Zhejiang Province China; 3 Department of Nursing, School of Medicine, Women’s Hospital, Zhejiang University, Hangzhou City, Zhejiang Province China; 4 Department of Nursing, Hangzhou Women’s Hospital, Hangzhou City, Zhejiang Province China; 5 Lawrence S. Bloomberg Faculty of Nursing, University of Toronto, Toronto, Canada

**Keywords:** Bedside case-based learning (BCBL), Bedside teaching, Case-based learning (CBL), Self-rating scale of self-directedness learning (SRSSDL), Critical thinking disposition inventory-Chinese version (CTDI-CV), Bachelor’s degree midwifery education

## Abstract

**Aim:**

This study aimed to investigate the impact of bedside case-based learning on critical thinking and capacity for self-directed learning in a group of midwifery students in China.

**Background:**

Bedside teaching is a well-established educational tool to improve the clinical practice of medical, nursing, and midwifery students. A new pedagogical approach; bedside case-based learning (BCBL) is an interactive teaching approach involving small-group, student-educator discussion to describe the etiology and management of a patient case. This new approach has been gradually integrated into Chinese midwifery education programs to promote clinical problem-solving skills, knowledge application, teamwork, and collaboration.

**Design:**

A quasi-experimental pre-test-post-test group design.

**Methods:**

This study used a quasi-experimental pre-test-post-test group design. A convenience sample of 67 third-year students majoring in midwifery at the university were prospectively enrolled in this study. Pre- and post-BCBL class surveys were conducted using the Self-Rating Scale of Self-Directed Learning (SRSSDL) and the Critical Thinking Disposition Inventory-Chinese Version (CTDI-CV) to assess changes in self-learning and critical thinking abilities before and after the intervention.

**Results:**

Following bedside case-based learning, the total scores for self-directed learning capacity and critical thinking increased, although there were no statistically significant differences (*P* > 0.05).

**Conclusions:**

Although there were no statistically significant differences between pre- and post-test results, students’ self-assessed scores in self-directed learning and critical thinking improved between pre- and post-test.

## Background

As a result of the 2016 ‘Two-Child Policy’ and the subsequent 2021 ‘Three-Child Policy’, China has increasingly faced the challenges of a midwifery workforce shortage [1]. The International Confederation of Midwives (ICM) has indicated that education is one of the three critical foundations for midwifery workforce development [2]. A 4-year direct-entry midwifery education for bachelor’s degrees was established in four universities in China in 2016 [3]. Teaching content in this program in the first two years is focused on fundamental theories. In the third year of the program, students complete courses relating to clinical practice in simulation lab. Students must complete at least ten months of continuous integrative clinical practicum in the fourth year of the program.

Bedside teaching is a vital component of midwifery education. Bedside Case-Based Learning (BCBL) combines interactive bedside teaching and case-based learning using instructor-led small group discussion to identify a presenting patient’s etiology and management. BCBL is a novel active learning approach that has been promoted as piquing student attention, motivating them to prepare ahead of time and work in groups, and enhancing their critical thinking skills, all of which are linked to their problem-solving abilities [4]. Although there are numerous studies on active learning strategies [5–7], the impact of a bedside, case-based learning approach to improve midwifery students’ self-directed learning and critical thinking skills has not been studied to date.

## Methods

### Study design

This study was a quasi-experimental design using one group pre-test-post-test design.

### Sample selection and setting

This study was conducted in an undergraduate direct-entry midwifery program at the Zhejiang Chinese Medical University nursing school in Zhejiang Province, China. A convenience sample of all midwifery students, having successfully completed foundational courses in Years 1 and 2; were provided with information about the study, a consent form, and invited to participate in the study. The study enrolled sixty-seven third-year students majoring in midwifery, which was the first-generation midwifery student at the Zhejiang Chinese Medical University nursing school.

Based on previous research and expert opinions, we chose a medium effect size (Cohen’s d = 0.5), targeting an 80% power and a 0.05 significance level. Using G*Power, we determined that 60 participants per group are needed. Allowing for a 10% dropout rate, we plan to recruit 66 students per group. These parameters were chosen to balance statistical power with practical resource constraints.

### Application of bedside case-based learning model

At the beginning of the semester, students completed the pre-class surveys; the Self-Rating Scale of Self-Directed Learning (SRSSDL), and the Critical Thinking Disposition Inventory (CTDI)-Chinese version to provide baseline data. At the end of the semester, all students were required to complete the post-class surveys with the same scales to evaluate the effect of BCBL in improving students’ learning abilities. The BCBL process lasted for the whole semester with a class held each week.

The BCBL approach was as follows:

Before class, clinical instructors prepared the teaching materials and required readings for the course, identified the learning objectives and the typical clinical scenario case one day in advance of class, collaborated with the identified patient on the process, and obtained consent from the patient. And clinical scenarios were found in real clinical wards in the hospital based on the different teaching themes to present key information related to patient history, symptoms, and initial findings. Before each class, students engage with the provided content and complete the required readings. There are a total of 14 themes over the semester (Table 1).

**Table 1:** Teaching themes in the BCBL approach to midwifery education

Teaching themes	
Gestational hypertension, Intrahepatic cholestasis of pregnancy	
Preterm birth & Post-term birth	
Peripartum cardiomyopathy	
Gestational diabetes mellitus	
Liver disease during pregnancy	
Anemia in pregnancy	
High-risk pregnancy	
Cesarean section	
Abnormal labor and delivery	
Shoulder dystocia	
Postpartum Hemorrhage	
Uterine rupture	
Amniotic fluid embolism	
Postpartum disorder	

Before beginning classroom activities, students are assigned to one of four parallel groups of seven to eight individuals. All clinical instructors received the same training on teaching content and how to deliver BCBL effectively in advance. The clinical instructors are midwives working at Hangzhou Women’s Hospital. Each class session begins with a brief overview of the topic and the class schedule.

Each clinical instructor presents a typical patient case with the same disorder using a slide deck. The clinical instructors then lead students to the bedside to directly acquire the patient’s medical history. Students then engage in small-group discussions under the supervision of the clinical instructor where they are encouraged to pose relevant clinical questions to patients and seek information using the Internet or the databases held by the hospital library. A student representative from each group presents a summary of the key findings to the class, the group’s responses to the questions raised, and clarification on any unanswered questions. Subsequently, the clinical instructors review the essential theoretical information and summarize it for the class. Students are required to complete and submit an assessment report at the end of each class. The assessment report includes the details of clinical case and the analysis of their own critical thinking.

### Instruments

Self-assessments completed by students included the Self-Rating Scale of Self-Directed Learning (SRSSDL) and the Critical Thinking Disposition Inventory-Chinese Version (CTDI-CV) to measure midwifery students’ self-directed learning ability, and critical thinking, at the beginning and end of the semester.

Williamson developed the self-rating scale of self-directed learning (SRSSDL) in 2007 [8]. The author authorized Shen Wangqin and Hu Yan [9] from Fudan University to translate it to Chinese in 2012. The SRSSDL consists of 60 items categorized into five broad areas, each consisting of 12 items. The categories included: awareness (understanding the factors that contribute to being self-directed learners); learning strategies (strategies recommended for being self-directed learners); learning activities (activities often used in self-directed learning); evaluation (attributes that help learners monitor their learning activities) and interpersonal skills (skills considered pre-requisite to becoming self-directed learners). The responses for each item are rated using a five-point Likert type scale (5 = Always, 1 = Never). The score ranges from 60 to 300 points, with a higher score indicating a higher ability for self-directed learning. The Chinese version of the SRSSDL has demonstrated good reliability and internal consistency (Cronbach’s α coefficient is 0.97).

Based on the California Critical Thinking Dispositions Inventory (CCTDI), Pang [10] adapted and updated the Critical Thinking Disposition Inventory-Chinese Version (CTDI-CV). The scale is a standardized 70-item, multiple-choice survey with seven dimensions: truth-seeking (10 items), open-mindedness (10 items), analyticity (10 items), systematicity (10 items), self-confidence (10 items), inquisitiveness (10 items), and cognitive maturity (10 items). The responses for each item are rated using a six-point Likert type scale (6 = Strongly Agree, 1 = Strongly Disagree). The total score ranges between 70 and 420 points with a higher score indicating higher critical thinking skills. The Chinese version of the CCTDI has demonstrated good reliability and internal consistency (Cronbach’s α coefficient is 0.90).

### Ethics approval

This study was approved by the Research Ethics Committee at the Nursing School of Zhejiang Chinese Medical University and conducted according to the Helsinki Declaration.

### Data analysis

The data were processed and analyzed using SPSS version 20.0 (Chicago, USA). Continuous variables are presented as mean ± standard deviation. Categorical variables are shown as the number of participants (percentage). A paired sample t-test was used to analyze the mean difference between pre- and post-test for the same students. P values < 0.05 were considered statistically significant.

## Results

### Participants

A total of sixty-seven third-year undergraduate students majoring in midwifery were enrolled in our study, representing a response rate of 100%. All participants were female, with an average age of 20.21 ± 0.729 years. They all completed the midwifery bedside teaching course with no study withdrawals. Most students (55/67) had indicated an interest in midwifery before admission, and 64/67 students wanted to work at the hospital after graduation (Table 2).

**Table 2:** Demographic information of the participants

Variable	*n*(%)
Age Group	
18 ~ 20 years	48(72)
21 ~ 25 years	19(28)
Majors of interest before admission	
Midwifery	55(82)
Other majors	12(18)
Work intention after graduation	
Midwifery and related work	64(96)
Other	3(4)

### Pre-and post-class self-directed learning

We compared the pre- and post-class survey scores of SRSSDL (Table 3). We found that the post-class scores for learning awareness, learning behavior, learning strategies, learning evaluation, and interpersonal skills of self-directed learning ability were no different when compared to the pre-class survey scores(*P* > 0.05).

**Table 3:** Pre-and post-class SRSSDL scores

Dimensions	Before BCBL	After BCBL	T value	*P* value
	Mean ± SD	Mean ± SD		
Awareness	27.36 ± 5.353	28.12 ± 4.819	0.835	0.405
Learning strategies	27.04 ± 5.221	27.12 ± 5.077	0.868	0.387
Learning activities	27.72 ± 5.564	28.17 ± 4.373	0.503	0.616
Evaluation	28.31 ± 5.980	28.67 ± 4.417	0.375	0.708
Interpersonal skills	26.99 ± 5.701	26.99 ± 5.820	0.279	0.781

### Pre- and post-class CTDI-CV scores

We compared the pre- and post-class survey scores of CTDI-CV and found no statistically significant difference between before and after the class (*P* > 0.05). (Table 4)

**Table 4:** Pre-and post-class CTDI-CV scores

Dimensions	Before BCBL	After BCBL	F value	*P* value
	Mean ± SD	Mean ± SD		
Truth-seeking	35.612 ± 6.543	36.817 ± 6.833	0.178	0.673
Open-mindedness	37.940 ± 6.483	37.400 ± 6.173	0.000	0.995
Analyticity	32.731 ± 7.959	34.017 ± 5.299	3.468	0.065
Systematicity	34.268 ± 6.195	35.467 ± 5.350	1.036	0.311
Self-confidence	32.328 ± 7.042	33.817 ± 5.537	2.365	0.127
Inquisitiveness	33.328 ± 6.702	35.800 ± 5.068	3.552	0.062
Cognitive Maturity	41.075 ± 8.459	41.567 ± 6.956	2.169	0.143

## Discussion

Midwifery is a practice-based specialty; with education programs aimed at cultivating skilled midwives with solid theoretical knowledge, proficient clinical practice skills, and the interpersonal skills required for caring for pregnant women and infants [11]. The four-year or five-year bachelor’s degrees; direct entry midwifery programs were re-established in four university nursing schools in 2016 after several years in abeyance [3]. Baccalaureate midwifery education is still developing in China in the face of a growing urgency to graduate increased numbers of midwives to meet demands under the recently implemented “Three-Child Policy”.

Historically, the midwifery education model was theory-based and taught in a traditional lecture-based format. However, such traditional models of midwifery education may no longer meet the needs of students. Students are reported to have often challenged in relation to participation and engagement in their own learning, difficulty integrating theory and practice, and a lack of ability to problem-solve in the clinical environment [12]. As a consequence, new graduates become comfortable with clinical work only when working at a hospital, with the development of critical thinking skills requiring immersion in authentic and/or simulated clinical settings and interaction with patients. Compared to the traditional training model, some studies have demonstrated the advantages of either CBL or bedside teaching [13–16]. Bedside case-based learning (BCBL) is a specialized form of interactive small-group teaching in the patient’s presence in the hospital and is reported to be a better way to integrate theoretical learning with clinical practice for midwifery students [4]. Previous studies have generally focused on either CBL *or* Bedside teaching as separate approaches. Furthermore, several studies have shown that the practice of bedside teaching has been declining in recent years for a variety of reasons, including an increased patient turnover secondary to reduced lengths of stay, concerns regarding patient privacy, and the higher costs associated with this intensive approach to teaching [13]. With the outbreak of COVID-19, simulation-based education replaced bedside teaching in health professions education [17]. Some studies have shown that the difference in knowledge and skills between non-bedside simulation teaching and bedside teaching is not significant [18–20]. The difference may lie in communication skills and abilities.

In this study, we combined bedside teaching and case-based learning in order to provide more interactive sessions. Our pre-post test design found that the scores for SRSSDL were not significantly different. One reason may be the small sample size with only sixty-seven participants. Although the scores did not show any significant difference in self-directed learning abilities pre- and post-class, students self-reported making progress in three dimensions: awareness, learning activities, and evaluation. The results also showed that students were acquiring new knowledge beyond the prescribed course objectives and raising questions independently.

Several studies are consistent with the results of our study. A study by Kulkarni et al. in 2019 reported highly positive results in the integration of case-based learning in bedside clinics [21]. Case-based leanring can be considered as a part of the learning strategies because they can help students become more autonomous and strategic in the learning process. Their study reported a high level of motivation among the students using this approach (88%). Dubey et al. and Nair et al. also reported similar results; showing 74–98% motivation among the students to learn using additional resources [22, 23]. Moreover, bedside teaching may be more engaging for students by presenting clinical case scenarios and allowing the students to solve problems the way they would as midwives working in wards. This BCBL approach is similar to the approach used by the United States Medical Licensing Exam (USMLE) in their final step 3 (Clinical Case Simulators).

Previous reviews have demonstrated that bedside teaching can improve the communication abilities of medical students and residents [14]. However, our study did not identify any significant improvement in students’ interpersonal skills. One possible reason may be that the number of people in each bedside teaching group is limited to ten. In addition, when taking a patient’s medical history, not every member of the group can engage in effective nurse-patient conversation. Students self-reported the lowest score in the domains of “share information with others” and “easy to work with others.” Clinical instructors may lack relevant teaching skills to encourage students to participate in teamwork. Additionally, the cultural aspects may also affect this outcome, A study [24] showed that Asian unique cultural heritage has shaped the learning styles of students who have been found to be passive or even silent learners in traditional classroom settings and who favor group cohesiveness and harmony.

Previous studies have shown that CBL is effective in teaching specialized nursing courses and increases students’ satisfaction and critical thinking skills [25]. Our study compared the pre-and post-class scores of each item in the Chinese version of the Critical Thinking Disposition Inventory (CTDI-CV) and found no statistical differences between pre-and post-class scores in each dimension. One possible reason may be that the clinical instructors themselves lack training in developing critical thinking skills in students. Another possible explanation is that the BCBL’s length and intensity were insufficient to produce meaningful benefits. Critical thinking skills develop over time and may require sustained and intensive effort. According to Glen [26], critical thinking is considered as an indispensable element of education and a trait of an educated person. The disadvantage is that one is unable to teach critical thinking if one is not a critical thinker oneself. Educators may not have their own critical and reflective thinking developed when they were learners, which makes it difficult for them to facilitate critical thinking as instructors. Approaches to developing those skills in educators may be an area that needs further exploration.

### Limitations

The outbreak of COVID-19, which resulted in visiting restrictions at hospitals, presented a serious limitation to further study. In addition, we did not include a control group with a standard teaching model for the analysis. Case-control studies with a larger sample size should be considered in future research to lessen the risk of confounding factors.

## Conclusion

In summary, a bedside case-based learning strategy for midwifery students did not lead to improvements in independent learning and critical thinking abilities in this study. This teaching method is also a challenge for clinical instructors; requiring excellent foundational knowledge, extensive clinical experience, and highly developed critical thinking skills. The new BCLC model may need more observations and further research to continually optimize and summarize past experiences and deficiencies for future improvement.

## Data Availability

The authors confirm that the data supporting the findings of this study are available within the article.

## Abbreviations

BCBL: Bedside Case-Based Learning
SRSSDL: Self-Rating Scale of Self-Directed Learning
CTDI-CV: Critical Thinking Disposition Inventory-Chinese Version
CBL: Case-Based Learning
ICM: International Confederation of Midwives
USMLE: United States Medical Licensing Exam
